# Supplementary material for: Over the Counter Pain Medications Used by Adults: A Need for Pharmacist Intervention
Source: Int J Environ Res Public Health. 2023 Mar 3;20(5):4505. doi: 10.3390/ijerph20054505 (PMC10001525; doi:10.3390/ijerph20054505)
Supplement: Supplementary file 1 [file ijerph-20-04505-s001.zip › ijerph-2204601-supplementary.pdf]

Assessing the safety of NSAIDs, paracetamol, and metamizole in the elderly

1. Age:
   ……….
2. Sex:
   - Female
   - Male
3. Education:
   - Primary
   - Secondary
   - Vocational
   - Higher
4. Place of residence:
   - Under 20 000 citizens
   - 20 000-100 000 citizens
   - 100 000-500 000 citizens
   - More than 500 000 citizens
5. How do you assess your economic situation?
   - Good
   - Average
   - Bad
6. Who do you live with?
   - with a partner
   - with children
   - with a caregiver
   - alone
   - in an assistance center
7. Please indicate your weight:
   …….
8. Please indicate your height (cm):
   .....
9. Do you use a cell phone?
   - Yes
   - No
10. Do you use a computer?
    - Yes
    - No
11. What does your physical activity look like?
    - I do not do any physical activity.
    - I do minimal physical activity such as gardening, cleaning, etc.

- 2.5 h of moderate exertion per week such as swimming, walking, dancing, gymnastics, and cycling.
- 5 h of moderate exercise per week

12. How often do you consume alcohol?
    - 2-3 times a week
    - Once a week
    - 1-2 times a month
    - Less than once a month
    - Not at all
13. How many cigarettes do you smoke?
    - One a day or more
    - Not at all
14. What chronic diseases do you suffer from?

Disease	Yes	No
Cardiovascular diseases (pulmonary embolism, heart failure, ischemic heart disease)	●	●
Hypertension	●	●
Diabetes	●	●
Kidney failure	●	●
Liver failure	●	●
Depression, neuroses	●	●
Epilepsy	●	●
Obesity	●	●
Cancer	●	●

15. Do you use dietary supplements?
    - Yes
    - No
16. If so, what supplements are you taking?
    - St. John's wort preparations
    - Preparations from ginkgo biloba
    - Preparations containing garlic
    - Preparations containing omega-3 acids
17. For what conditions do you take NOAs (Non-opioid analgesics) drugs?

Dolegliwość	Yes	No
Headache, toothache, joint pain, muscle pain, post-traumatic pain	●	●
Fever, cold, influenza	●	●
Rheumatoid arthritis, osteoarthritis	●	●
Prevention of myocardial infarction, embolic and thrombotic complications, angina pectoris	●	●
Gout	●	●

18. What oral analgesics, antipyretics do you use, how often and with whose prescription?

	Never	Several times a week	Several times a month	Less than once a month	Doctor or pharmacist
Acetylsalicylic acid (Aspirin)	● Yes<br>● No	● Yes<br>● No	● Yes<br>● No	● Yes<br>● No	● Yes<br>● No
Paracetamol (Apap, Panadol,)	● Yes<br>● No	● Yes<br>● No	● Yes<br>● No	● Yes<br>● No	● Yes<br>● No
Ibuprofen (Ibuprom, Ibum, Neurofen)	● Yes<br>● No	● Yes<br>● No	● Yes<br>● No	● Yes<br>● No	● Yes<br>● No
Ketoprofen (Ketonal, Profenid,)	● Yes<br>● No	● Yes<br>● No	● Yes<br>● No	● Yes<br>● No	● Yes<br>● No
Naproxen (Naproxen, Aleve, Anapran)	● Yes<br>● No	● Yes<br>● No	● Yes<br>● No	● Yes<br>● No	● Yes<br>● No
Nimesulide (Nimesil, Minesulin)	● Yes<br>● No	● Yes<br>● No	● Yes<br>● No	● Yes<br>● No	● Yes<br>● No
Diclofenac (Diclac, Majamil, Voltaren)	● Yes<br>● No	● Yes<br>● No	● Yes<br>● No	● Yes<br>● No	● Yes<br>● No
Metamizole (Pyralgina, Gardan)	● Yes<br>● No	● Yes<br>● No	● Yes<br>● No	● Yes<br>● No	● Yes<br>● No
Indomethacin (Metindol)	● Yes<br>● No	● Yes<br>● No	● Yes<br>● No	● Yes<br>● No	● Yes<br>● No

19. What adverse reactions did you experience after using the drug?

	Acetylsalicylic acid (Aspirin)	Paracetamol (Apap, Panadol,)	Ibuprofen (Ibuprom, Ibum, Neurofen)	Ketoprofen (Ketonal, Profenid,)	Naproxen (Naproxen, Aleve, Anapran)	Nimesulide (Nimesil, Minesulin)	Diclofenac (Diclac, Majamil, Voltaren)	Metamizole (Pyralgina, Gardan)	Indomethacin (Metindol)
Allergic reactions (dermatidis, urticaria, pruritus)	•	•	•	•	•	•	•	•	•
Liver dysfunction	•	•	•	•	•	•	•	•	•
Renal dysfunction	•	•	•	•	•	•	•	•	•
Dizziness and tinnitus	•	•	•	•	•	•	•	•	•
Prolonged bleeding time	•	•	•	•	•	•	•	•	•
Gastrointestinal disorders (heartburn, vomiting, abdominal pain)	•	•	•	•	•	•	•	•	•
Ulcers of the stomach or duodenum	•	•	•	•	•	•	•	•	•
Bleeding from the gastrointestinal tract	•	•	•	•	•	•	•	•	•

20. Do you use painkillers or/and anti-inflammatory medications on the skin (Ketoprofen Ziaja, Opokan Actigel, Voltaren max, Olfen), if yes, from whose prescription?

	Yes	No
Never	•	•
Daily	•	•

Several times a week	●	●
Several times a month	●	●
Less than once a month	●	●
Doctor	●	●
Pharmacist	●	●

21. Have there been any side effects while using medications on the skin?

	Yes	No
Local skin reactions: rash, eczema, erythema, pruritus	●	●
Photosensitivity	●	●
Gastrointestinal disorders (heartburn, indigestion, vomiting, abdominal pain)	●	●
Exacerbation of already existing renal failure	●	●

22. Did you report any adverse reactions to the drug?
    - Yes
    - No
23. Did you know about the possibility of reporting adverse reactions?
    - Yes
    - No
24. In what form and to whom did you happen to report them?
    - I self-reported/reported to the Adverse Drug Reactions Monitoring Department
    - I reported it to the doctor, pharmacist, nurse
25. What recommendations did the doctor make after being informed of persistent side effects?
    - Discontinuation of the medicine
    - Another drug was prescribed
    - Continuing therapy with no change
26. When prescribing any of the mentioned pain medications, did the doctor ask about chronic diseases?

- Yes and therefore gave me additional information on the safety of use or modified the therapies, e.g., switched tablets to gel.
- Yes and ordered additional monitoring for adverse effects (measuring blood pressure, urine tests, more blood glucose control).
- The doctor has not asked about this.

27. Have you ever suffered from gastric or duodenal ulcer disease?
    - Yes
    - No
28. Did your doctor prescribe you any gastric "shielding" medications while you were being treated with painkillers?
    - Yes
    - No
29. Have you used two or more pain medications at the same time?
    - Yes
    - No
30. Where was the drug acquired?
    - Pharmacy
    - Other
31. Where do you get your information on how to take your medications?
    - From a doctor/nurse/ paramedic
    - From a pharmacist
    - From family/friends
    - Media
32. Do you have a vision impairment?
    - Yes
    - No
33. Do you read the leaflet that accompanies the medicine package?
    - Yes
    - No
34. Do you follow the recommendations on the leaflet?
    - Yes
    - No
35. Do you sometimes forget to take your medication?
    - Yes
    - No
36. What happens when you forget to take your medication at a particular time?
    - I skip the dose that I have forgotten to take.
    - I take it when I have remembered to take it.
    - I take a double dose the next time I should take medication.
37. Do you take care to take your medications at appropriate times?
    - Yes
    - No
38. In what way?
    - I remember

- A family member/alarm/organizer/app helps me.

39. Have you heard of the "envelope of life"?
    - Yes, and I own one at home.
    - Yes, but I do not have one prepared.
    - No
40. What do you sip your pills with?
    - Water
    - Other (coffee/milk/juice)
41. How do you take your pain medication?
    - Before eating, on an empty stomach
    - During eating
    - After eating
42. Do you have a separate storage area for your medications?
    - Yes
    - No
43. Do you check the expiration date of medications stored at home before taking them?
    - Yes
    - No
44. Do you know that the same drugs (same active substance) can have different trade names?
    - Yes
    - No
